# Oxidative Stress Biomarkers in the Shrimp *Macrobrachium amazonicum* (Heller, 1862): Assessment in an Environmental Preservation Area in the Brazilian Amazon

**DOI:** 10.1002/tox.70074

**Published:** 2026-03-07

**Authors:** Natália Eduarda da Silva, Arllon José dos Santos Dias, Layane Vitoria dos Santos e Santos, Natalie de Matos Araujo, Darlan Coutinho dos Santos, Jefferson Romáryo Duarte da Luz, Gabriel Araujo‐Silva

**Affiliations:** ^1^ Organic Chemistry and Biochemistry Laboratory Amapá State University (UEAP) Macapá Amapá Brazil; ^2^ Postgraduate Program in Biodiversity and Biotechnology of the BIONORTE Network (PPG‐BIONORTE) Federal University of Amapá (UNIFAP) Macapá Amapá Brazil; ^3^ Federal Institute of Education, Science and Technology of Amapá (IFAP) Macapá Amapá Brazil; ^4^ College of Biological Sciences Federal University of Amapá (UNIFAP) Oiapoque Amapá Brazil

**Keywords:** Amazon River, biological marker, crustacean, oxidative inhibitor, pollutant agent

## Abstract

Anthropogenic activities generate a significant amount of pollutants that are released into the environment, causing physiological and ecological disturbances. Among the xenobiotics present in aquatic ecosystems, numerous chemical and organic compounds have oxidative potential or are metabolized through oxidative processes, which may amplify the damage caused by reactive oxygen species to biological systems. The quantification of cellular damage and antioxidant defenses can be used as biomarkers for early aquatic contamination. The aim of this research was to use the shrimp *Macrobrachium amazonicum* as a bioindicator species to assess oxidative damage caused by xenobiotics in an Environmental Protection Area in the Brazilian Amazon. The analyses evaluated physicochemical parameters, Iron, Copper, Total Solids, pH, and Temperature, as well as non‐enzymatic and enzymatic oxidative stress biomarkers: Thiobarbituric Acid Reactive Substances, glutathione (GSH), and catalase (CAT) in hepatopancreas homogenates. Among the analyzed metals, only copper (Cu) showed a statistically significant influence on GSH and CAT activities, whereas the other parameters did not exhibit significant effects. Oxidative stress parameters can be important tools in biomonitoring work, helping to understand the effects of contamination on aquatic organisms and providing important information on cellular defense modulations.

## 1 Introduction

Antioxidant defenses have the ability to be activated or diminished by pro‐oxidant agents, depending on the duration and intensity of exposure. Furthermore, cellular barriers provide important protection against exposure to particles [1]. At the same time, alterations in these defenses are associated with various categories of xenobiotics, disparities in sensitivity between species, and the influence of environmental and biological factors [2].

Organisms exposed to trace metals can be induced to produce Reactive Oxygen Species (ROS), which causes oxidative stress, resulting in various harmful effects on cells [3] and DNA damage [4]. These species may include hydrogen peroxide, superoxide radical, and hydroxyl radical. Additionally, contamination by pesticides can also cause oxidative damage to aquatic species. These xenobiotics enter aquatic environments through leaching and runoff from agricultural areas and can remain there for months or even years [5]. They can trigger the production of ROS through various biochemical mechanisms, such as disruption of electron transport across the cell membrane, facilitation of the Fenton reaction, inactivation of antioxidant enzymes, and depletion of free radical scavengers [6].

Aquatic organisms respond to environmental changes and can be considered efficient bioindicators of water quality [7]. Therefore, research often adopts species for pollution analysis. A bioindicator should accumulate high levels of pollutants, be restricted to a specific location to better expose local pollution, be abundant in the area, have a long lifespan, provide suitable tissue for future research, sampling, and easy collection, occupy an important position in the food chain, and have a good dose‐effect relationship. Aquatic ecosystem bioindicators are the most commonly used to provide information on water quality [8, 9].

Crustaceans, especially the shrimp *Macrobrachium amazonicum* (Heller, 1862), commonly known as the regional shrimp [3], is an abundant native species with considerable commercial and ecological importance in northern Brazil. Its benthic lifestyle increases exposure to sediment‐associated contaminants, while its antioxidant defense system provides sensitive biomarkers of environmental stress [8]. Although this species has been proposed as a potential bioindicator, few studies have explored its biomarker responses in natural ecosystems, particularly within legally protected areas [10, 11]. This study is among the first to assess oxidative stress biomarkers in *M. amazonicum* from an Environmental Protection Area (EPA) in the Amazon, integrating physicochemical parameters with enzymatic and non‐enzymatic responses to better understand contamination effects and support freshwater biomonitoring strategies.

The antioxidant defense system in shrimp is a set of biochemical mechanisms that protect cells against damage caused by reactive oxygen species, which can be generated in response to the presence of environmental contaminants [4]. This system includes antioxidant enzymes such as SOD, CAT, and GPx [5], as well as antioxidant or non‐enzymatic molecules like GSH, which work together to neutralize free radicals and ROS.

Shrimps, due to their benthic habit, have a higher likelihood of coming into contact with pollutants, as sediments are considered sinks for a range of environmental contaminants [12, 13]. Therefore, shrimp may increase the activity of these enzymes and the levels of GSH as an adaptive response to oxidative stress induced by pollutants. However, in cases of severe or prolonged contamination, this system may become overwhelmed, leading to oxidative damage and negative impacts on the health of the shrimp and the aquatic ecosystem [14]. Thus, the evaluation of the antioxidant system provides important information about exposure to contaminants and environmental health [15].

Aquatic ecosystems in environmental conservation units are vulnerable, mainly due to the transport of pollutants from unsustainable industrial and agricultural activities in the surrounding areas [16]. Environmental Protection Areas (EPAs) are generally large areas with some degree of human occupation, characterized by abiotic and biotic attributes, and their objectives are to protect biological diversity, regulate the process of occupation, and ensure the sustainability of natural resource use [17]. In this context, the purpose of this study was to use *M. amazonicum* as a bioindicator species to assess oxidative damage caused by xenobiotics in an EPA in the Amazon/Brazil and to evaluate the correlation between the presence of xenobiotics and the biomarkers assessed.

## 2 Materials and Methods

### 2.1 Sampling Points

The water and shrimp samples were collected during the wet season in the months of March 2022 (0°03′17.9″ S 51°07′32.3″ W), April 2022 (0°03′18.8″ S 51°07′34.7″ W), and May 2022 (0.05526505″ S 51.12779665″ W) which corresponds to the period of greatest hydrological flow and contaminant mobilization in the Amazon basin in the EPA known as “Fazendinha,” located in the state of Amapá (AP), in the Amazon region of Brazil (Figure 1). This area is situated between the “Fazendinha” district in the municipality of Macapá (AP) and the “Fortaleza” district in the municipality of Santana (AP), bordered to the south by the Amazon River. The “Fazendinha” EPA was selected as the sampling site because it represents a transition zone between urbanized areas and relatively conserved Amazonian environments. This area receives diffuse inputs from nearby agricultural and urban activities, making it a relevant site for assessing biomarkers of environmental contamination in resident species. All sampling points were located within the EPA to capture spatial and temporal variability inside this protected area. No external reference site was used, as the objective was to investigate biomarker responses within the EPA itself rather than establish comparisons with a pristine control location.

**FIGURE 1:**
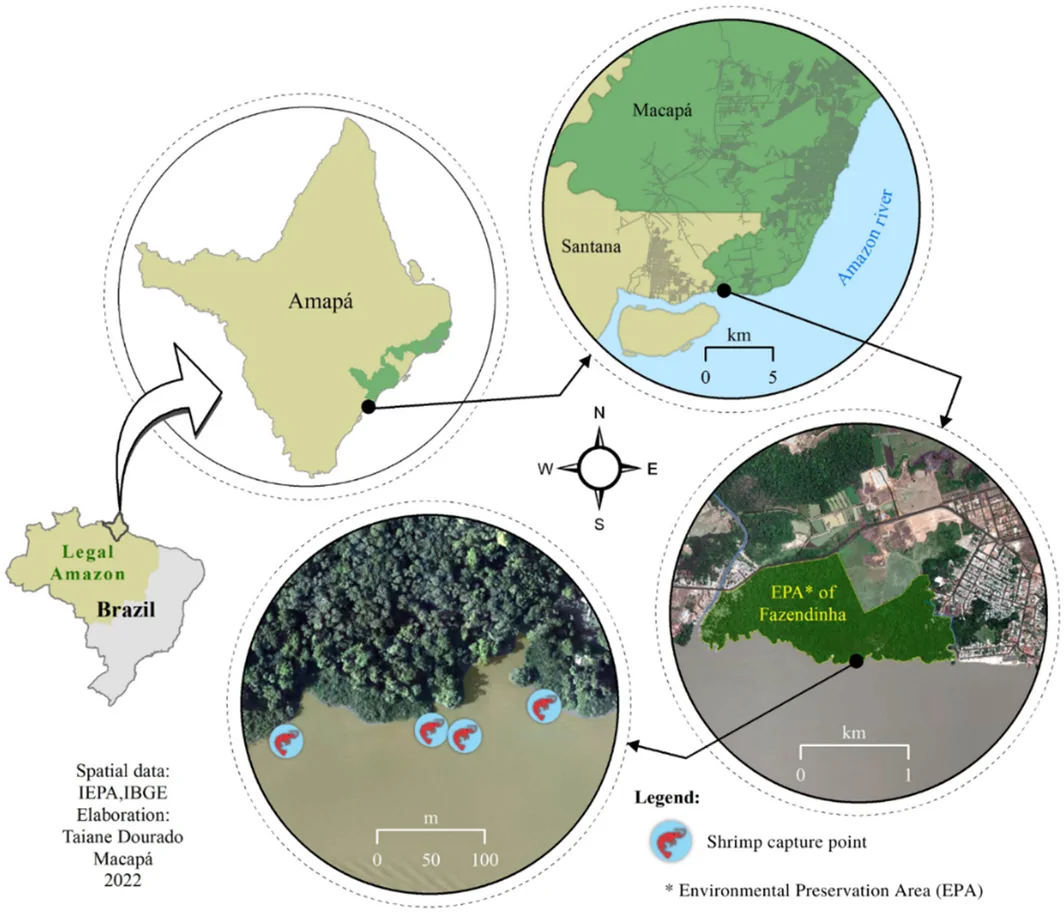
Location of water sampling points and shrimp capture in the “Fazendinha” Environmental Protection Area in the municipality of Macapá‐AP/Amazon/Brazil.

### 2.2 Water Analysis

Water samples were stored in sterile polyethylene bottles with a capacity of 7 L, collected from 3 points. The sampling procedure was carried out at the water surface. At each sampling site, three independent water samples were collected during each sampling campaign to provide replication and capture spatial heterogeneity. The replicate samples were processed and analyzed separately for physicochemical and metal parameters, and the mean values were used for statistical analyses. For analysis, the procedure was based on Resolution No. 357/2005 of the National Environmental Council (CONAMA/Brazil), which establishes the reference values permitted in Class II rivers, which are for drinking water supply after conventional treatment, protection of aquatic communities, primary contact recreation such as swimming, water skiing, and diving (according to Resolution CONAMA No. 274, 2000), transparency of vegetables, fruit plants, parks, gardens, sports fields, and recreational areas with which the public may have direct contact, and aquaculture and fishing activities, according to the resolution.

### 2.3 Total Solids

The total solids (STD) analysis was performed by adding a 10 mL aliquot of water into pre‐weighed crucibles, with the procedure being done in triplicates. This method was based on Nunes et al. [18]. The samples were kept in an analog sterilization and drying oven (My Labor equipment, SSA—40 L, São Paulo, Brazil) at 105°C. After the evaporation of the samples, the crucibles were cooled to room temperature in a desiccator and weighed, with the final weight subtracted from the initial weight.

### 2.4 pH and Temperature

The pH and water temperature analyses were conducted using the Hanna Multiparameter Probe (Hanna Instruments, HI 9892, Eden Way, England), according to the manufacturer's instructions.

### 2.5 Iron (Fe) and Copper (Cu) Analysis

For the analysis of Fe and Cu, the methodology described by [19] was used. Briefly, 0.25 g of phenanthroline was dissolved 0.25 g in 100 mL of ethanol. The water samples were analyzed from the transfer of a 25 mL aliquot of the sample, then 1 mL of ascorbic acid solution (1% (m/v)) was added, with the addition of 4 mL of the ascorbic acid solution. 1,10‐phenanthroline and 5 mL of pH 4.5 buffer solution, measuring the absorbances of the complexes at 511 nm for Fe and 371 nm for Cu in a UV–VIS Spectrophotometer (BEL Photonics—UV—M51, Monza (Milano), Italy), using a reagent blank, which was prepared in the same way as the solutions of work without the addition of analytes.

### 2.6 Collection, Morphological Analysis and Processing of Shrimp Hepatopancreas Homogenate

The shrimp (93) were selected based on weight (between 5 and 8 g), according to the data from the first and third quartiles of the total sample, and were collected using the “Matapi” fishing technique. While still alive, they were subjected to biometrics. Then, they were euthanized by hypothermia to preserve the tissue [20]. The collections followed Brazilian legislation, with authorization from the Chico Mendes Institute for Biodiversity Conservation (ICMBio/SISBIO—Authorization: 50376‐2).

The preparation of tissue homogenates involved the process of adding potassium phosphate buffer with pH = 7.4 [21], with the hepatopancreas being previously macerated in a cooled mortar and pestle. After maceration, the pools were subjected to centrifugation at 4000 rpm for 10 min to separate the tissue impurities, resulting in the so‐called crude homogenates at a ratio of 1:10 [22]. Aliquots of these homogenates were then used to analyze the activities of Catalase (CAT), Glutathione (GSH), Proteins, and to determine Lipoperoxidation (LPx).

Metal analyses were restricted to water samples. Tissue metal concentrations in *M. amazonicum* were not determined in this study due to logistical and financial constraints during the sampling campaigns.

### 2.7 Assay of Lipid Peroxidation Products

Lipid peroxidation was evaluated through the determination of Thiobarbituric Acid Reactive Substances (TBARS), with modifications [23]. The concentration of TBARS was obtained by colorimetric determination with absorbance at 535 nm and expressed in nmol/g protein. This assay quantifies malondialdehyde (MDA), an alkylating agent derived from the degradation of polyunsaturated lipids by ROS [24].

### 2.8 Reduced GSH Content

The concentration of reduced GSH was analyzed using the methodology proposed by [25]. The absorbance was measured in a UV–VIS spectrophotometer at 420 nm, and the GSH concentration was expressed in μmol/g protein.

### 2.9 CAT Assay

The CAT activity in the hepatopancreas homogenate was determined by spectrophotometry [26]. Briefly, the diluted samples were mixed with the reaction mixture (0.03% H_2_O_2_ dissolved in 1 M Tris/5 mM EDTA, pH 8.0), and the absorbance was measured at 240 nm using a spectrophotometer. The enzymatic activity was expressed in U/g protein.

### 2.10 Protein Determination

Protein determination was performed using a BIOCLIN kit, which corresponds to a colorimetric test, following the manufacturer's instructions. The sample and standard readings were conducted at a wavelength of 545 nm in the spectrophotometer, and the enzymatic activity was normalized by the total protein content.

### 2.11 Statistical Analysis

All results were tested for normality and homogeneity of variance using the Shapiro–Wilk and Levene tests, respectively. One‐Way ANOVA with Greenhouse–Geisser correction and Student's *t*‐test using the GraphPad version 6.0 software were conducted to investigate any significant differences within and between data sets. The significance criterion was set at *p* < 0.05. In addition to univariate analyses, Pearson's correlation tests were performed to assess relationships between metal concentrations and oxidative stress biomarkers (GSH and CAT). These analyses aimed to explore potential associations between environmental exposure and physiological responses in *M. amazonicum* . Correlations with *p* < 0.05 were considered statistically significant.

## 3 Results and Discussion

### 3.1 Analysis of the Physical–Chemical Parameters of Water

The EPA is located near the Igarapé da Fortaleza, a water body that suffers various environmental degradations, originating from the discharge of minerals, which has been considered one of the main environmental pollution problems in the Amazon region [27]. Additionally, it is close to the Fazendinha bathing area, which is affected by anthropogenic actions degrading its bathing water quality due to the increasing production of solid and liquid waste generated by users and, consequently, released into the environment [28].

The hydrogen potential of the water varied from 5.8 to 6.5, which are values characteristic of the Amazon River during the rainy season, as it tends to have more acidic pH values. These results are also corroborated by the study of [29], who measured pH levels in the waters of the Amazon River along the city of Macapá's waterfront (AP) during the rainy season, with similar results for water temperature (pH = 6.00–6.31 and temperature = 26°C and 27°C).

Trace metals, such as zinc, copper, and iron, play a biochemical role in the vital processes of all aquatic plants and animals and are important in the aquatic environment in trace amounts [30]. Regarding the analysis of Fe and Cu concentration in the collected water, represented by mg/L, the results show a statistically significant difference (*p* = 0.0059) when comparing the Fe values between March and May (1st and 3rd collection) as shown in Figure 2A. For Cu, there was also a statistically significant difference (*p* = 0.0389) between April and May (2nd and 3rd collection), and also a statistically significant difference (*p* = 0.0011) between March and May (1st and 3rd collection), these results can be seen in Figure 2B. Throughout the three periods, the concentration of Fe remained within the standards established by the resolutions, while for Cu, the values exceeded the limits (Table 1).

**FIGURE 2:**
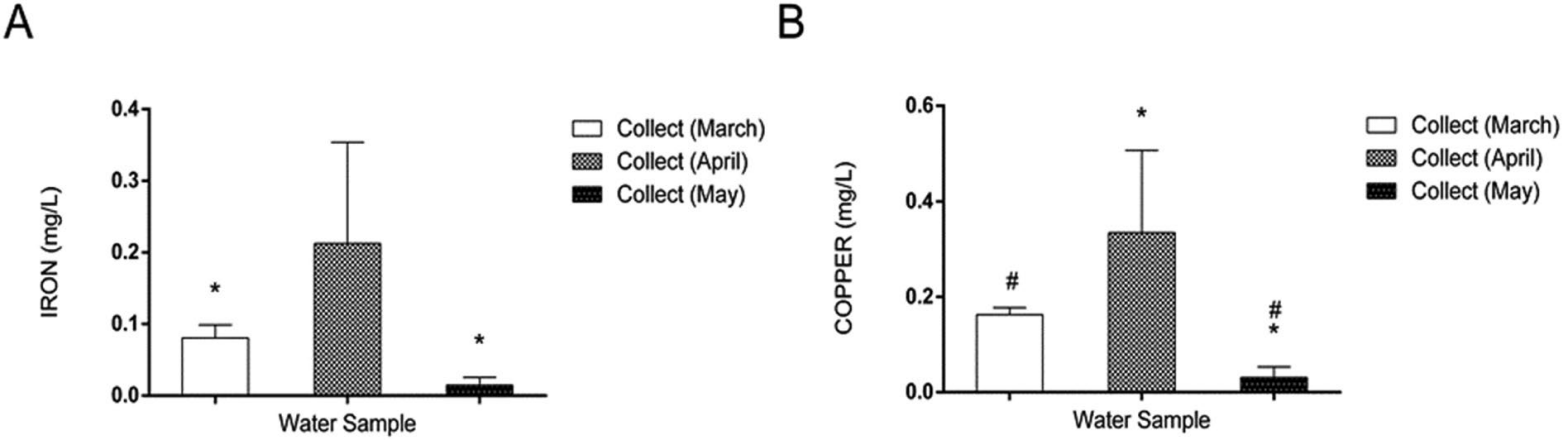
Water quality parameters—Iron (Fe) (A) and Copper (Cu) (B) in mg/L. * indicates a statistical difference between the March and May collections for Fe (A). * indicates a statistical difference between the April and May collections for Cu, and # indicates a statistical difference between the March and May collections for Cu (B). For all analyses, the unpaired Student's *t*‐test was performed, and *p* < 0.05 was considered statistically significant.

**TABLE 1:** Metal concentration (means and standard deviations, in mg/L) in water samples from the “Fazendinha” Environmental Protection Area in the municipality of Macapá‐AP/Amazon/Brazil.

Metals	Months			CONAMA (2005)^a^
	March	April	May	
Fe	0.0805 ± 0.018	0.2123 ± 0.1413	0.0144 ± 0.0111	0.300^a^
Cu	**0.1621 ± 0.0152**	**0.3343 ± 0.1721**	**0.0308 ± 0.0226**	0.009^a^

*Note:* Bold values indicate concentrations exceeding the maximum limits established by Brazilian legislation (CONAMA Resolution 357/2005, Class 2).

^a^ Maximum value allowed by Brazilian legislation (Class 2, Resolution 357/2005) of the National Environmental Council (CONAMA, 2005).

A common example of toxicity due to excess metal is the Fenton reaction. In this reaction, ferrous ions produce hydroxyl radicals and other reactive oxidizing substances through the decomposition of hydrogen peroxide (H_2_O_2_) [31]. Additionally, metals with multiple valence states (such as copper (Cu): Cu(I)/Cu(II)) can also electrochemically react with H_2_O_2_ in Fenton‐type reactions, forming radical ion species [32]. Such radicals can rapidly damage proteins, lipids, and nucleic acids [33]. These metals can accumulate in aquatic life, enter the food chain, and cause serious health damage to humans when contamination and exposure are significant [34].

The high presence of metal ions is considered a factor of imbalance in aquatic environments, as it can directly participate in redox reactions that produce ROS, sequestering or inactivating molecules involved in antioxidant defense systems [35]. This suggests that their disparity may reduce the activities of endogenous antioxidant defense enzymes. In addition to the significant presence of metal ions, it is important to highlight that other xenobiotics also play a relevant role in generating oxidative damage in aquatic environments [36]. These substances, foreign to the environment, are usually introduced into the water through various point and non‐point sources, including the leather industry, coal mining, agricultural activities, and domestic waste [37]. It is worth noting that contamination of crustaceans by Fe is common when the environment exhibits an abundance of this micronutrient [38].

Metals such as cobalt, iron, nickel, magnesium, copper, manganese, zinc, chromium, and selenium play functional roles in the body and are essential trace elements for various physiological and biochemical functions [39]. Additionally, in acidic waters, studies on the Nyamwamba River have shown an increase in Cu concentration [40]. Copper can be found in two different oxidation states in the environment [41] mainly in suspended particles in rivers [42, 43].

Aquatic organisms absorb essential metals from water, sediment, or even food for normal metabolism [44]. However, depending on the concentration level of metals in the environment, there can be a decrease in growth, reproduction [45], and population size of a given species. Excessive ingestion can produce toxic effects [46]. Studies on *Sparus aurata* species showed that metals like Cu and Cd posed the greatest potential health risk [47]. Both the presence of metal ions and exposure to xenobiotics represent serious challenges to the quality and health of aquatic ecosystems. Therefore, active monitoring with bioindicators should be used as a complement to physicochemical analyses. Biomonitoring through a comprehensive understanding of biomarkers can serve as health measures in freshwater environments [48].

For STD, a statistical difference (*p* = 0.0298) was observed between the collection times (Figure 3), and this finding is corroborated by the fact that Amazon rivers present high levels of organic matter in their waters, which occurs due to natural processes involving the biome [49]. This factor may justify the values obtained in this study, but it is worth noting that the results found are below the values established by the current legislation, [50] CONAMA 357/2005.

**FIGURE 3:**
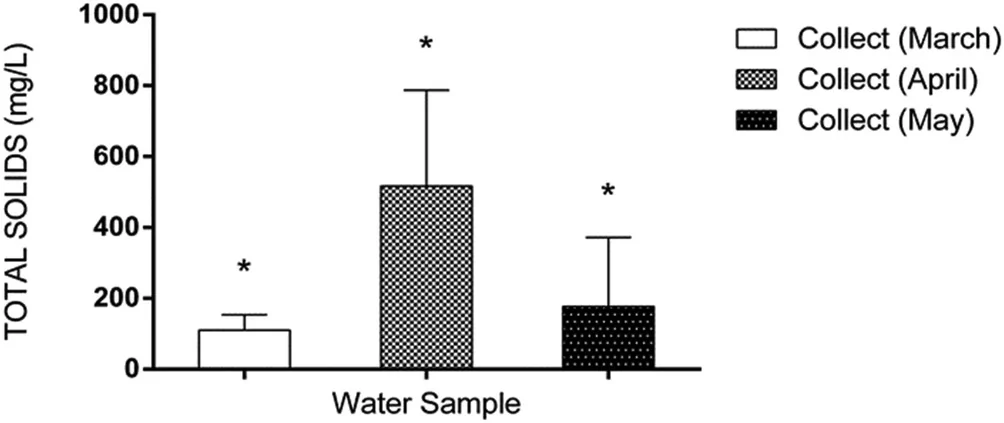
Water quality parameters (Total Solids) collected in March, April, and May 2022 in the “Fazendinha” Environmental Protection Area in the municipality of Macapá‐AP/Amazônia/Brazil. * indicates statistical difference between March, April, and May collections for One‐Way ANOVA. *p* < 0.05 was considered statistically significant.

### 3.2 Morphological Observations of the Shrimp Collected

The results related to the shrimp morphology did not show any statistical difference (Figure 4), which was expected due to the use of shrimp weighing between 5 and 8 g, in order to use a smaller number of shrimp in the preparation of the pools, thus minimizing the impact of removing this species from its habitat.

**FIGURE 4:**
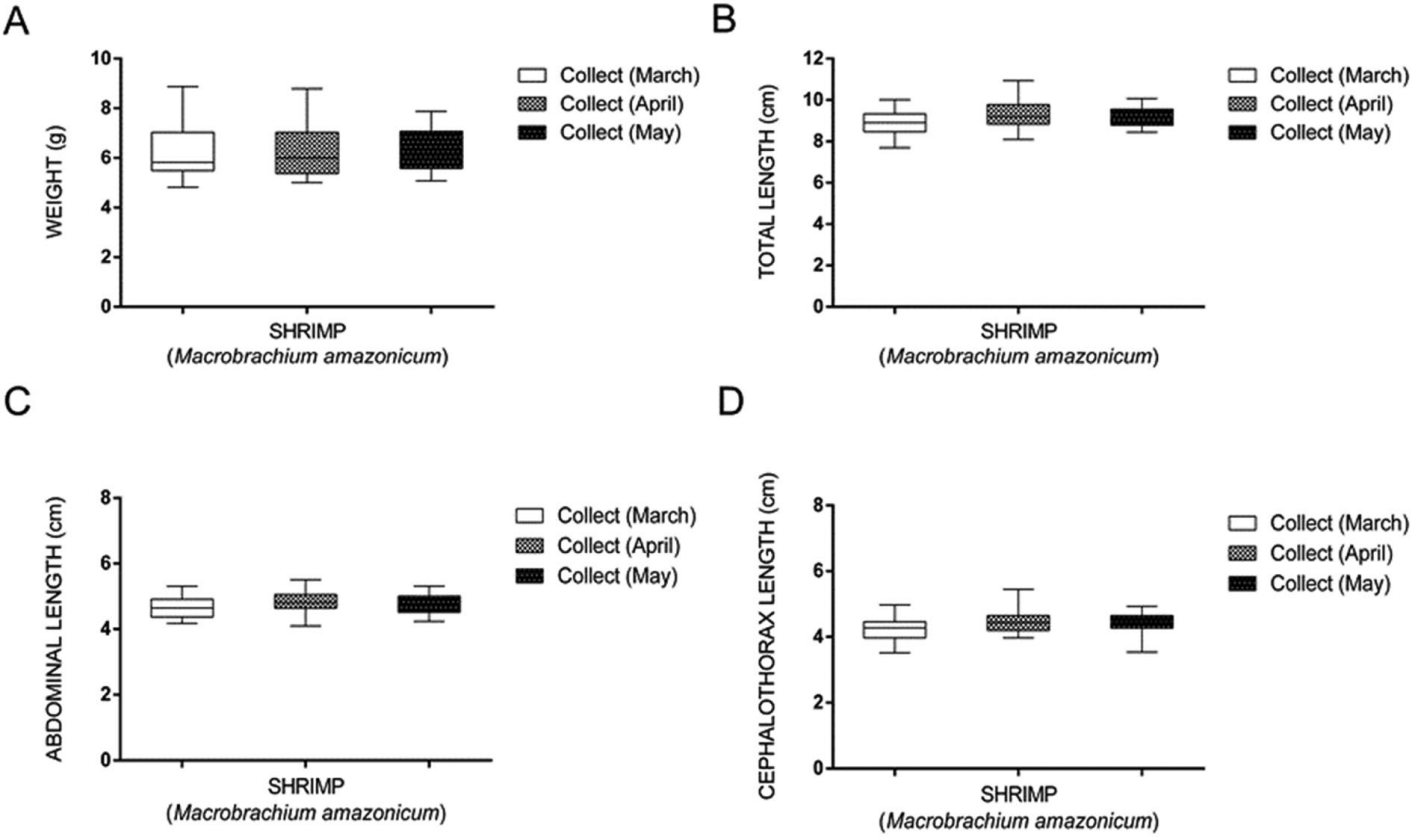
Morphological observations of shrimp collected in March, April and May 2022 in the “Fazendinha” Environmental Protection Area in the municipality of Macapá‐AP/Amazônia/Brazil. No statistically significant differences were observed for Weight (A), total length (B), abdominal length (C), and cephalothorax length (D).

### 3.3 Oxidative Stress Biomarkers

Biomarkers identify early warning signs of damage to organisms and can effectively complement chemical and ecological approaches to assess the state of water quality [51]. For the analysis of oxidative stress biomarkers (Figure 5) in *M. amazonicum* , the hepatopancreas (Figure 5) was used, as it is involved in metabolism, immune functions, nutrient absorption, and detoxification of xenobiotics, being one of the main organs affected by environmental stressors [52]. The accumulation of heavy metals in aquatic animals promotes the production of ROS, inhibits antioxidants, and, as a result, negatively impacts the immune system [47]. The correlation between Cu concentrations and the analyzed antioxidant biomarkers revealed distinct response patterns throughout the sampling period. It was observed that GSH showed greater sensitivity to Cu variation, with positive correlations in all months and a highly significant value in April (*r* = 0.99; *p* = 0.0228). On the other hand, the correlations between Cu and CAT activity were weak and not significant in all analyzed months (*r* ranging from −0.63 to 0.46).

**FIGURE 5:**
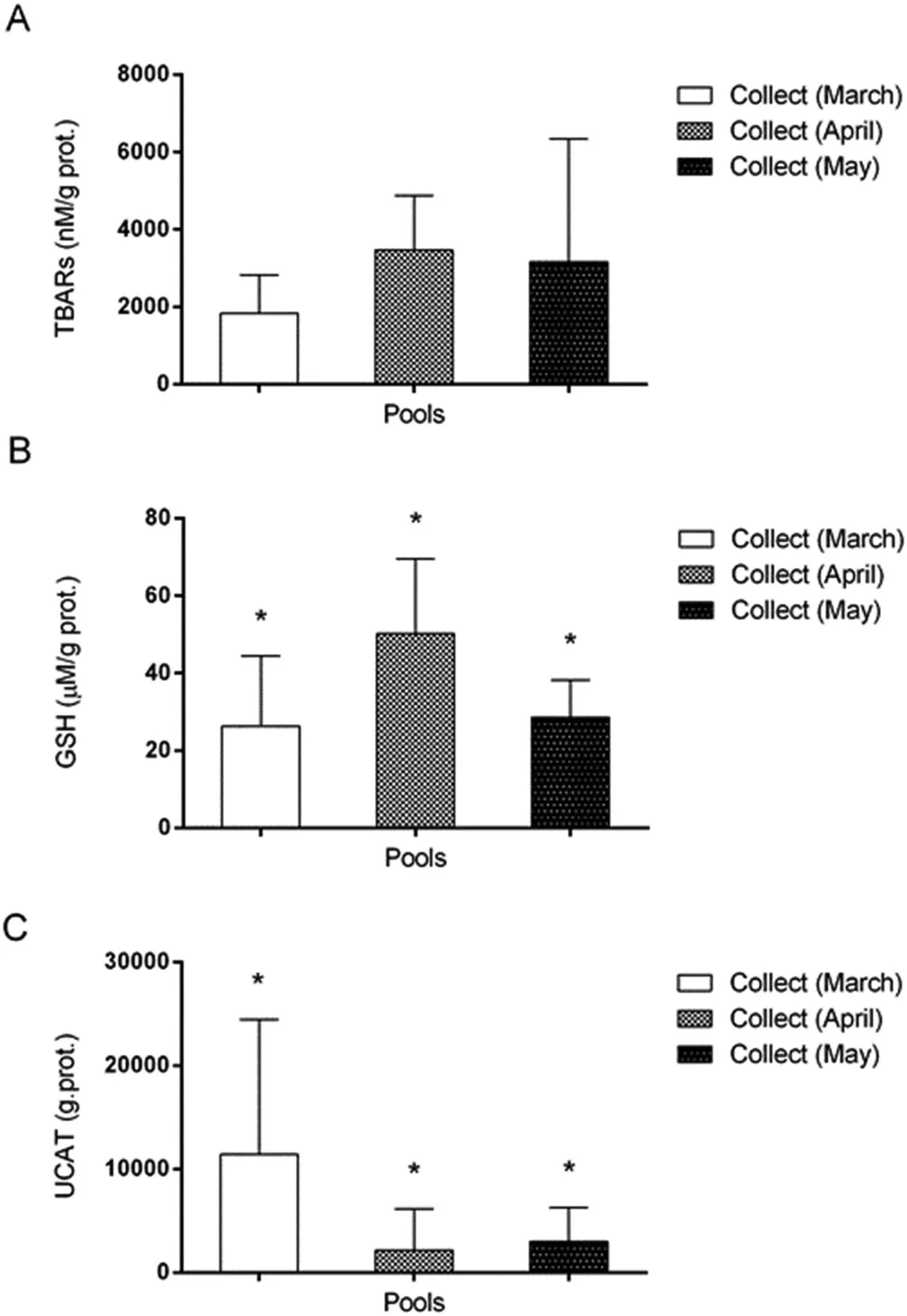
Oxidative stress biomarkers—TBARs (A), GSH (B), and CAT (C) in the shrimp hepatopancreas homogenate at the three collection times (March, April, and May 2022) in the “Fazendinha” Environmental Protection Area in the municipality of Macapá‐AP/Amazônia/Brazil. * indicates statistical difference between.

Thiobarbituric acid reactive substances are the most widely used assay for lipid peroxidation and serve as a good biomarker for studies involving pesticides and heavy metal pollution in aquatic organisms [53], with MDA being one of the products of the reaction. The formation of MDA is directly associated with lipid peroxidation, a process that occurs when organisms or cell membranes are exposed to a significant level of oxidative stress, often caused by harmful environmental pollutants, and it can be considered the most commonly used biomarker to measure the level of oxidative stress [54]. Therefore, elevated detection of MDA indicates considerable oxidative damage and serves as an early warning signal for an aquatic environment that has experienced substantial contamination impacts. The correlation between lipid peroxidation and MDA with the assessment of environmental contamination lies in the fact that the presence of toxic pollutants, such as heavy metals or organic chemicals, can trigger an increase in ROS production in aquatic organisms, providing valuable insights into the impacts of pollution and the quality of aquatic ecosystems [55]. However, the results did not show a significant increase in the levels of lipid peroxidation products (Figure 5A), similar to a study conducted with the crustacean *Aegla singularis* [56] and the species *Aegla castro* [57].

Catalase is a crucial enzyme that plays an essential role in the antioxidant defense of organisms by accelerating the decomposition of H_2_O_2_ into water and oxygen [58]. The relationship between CAT activity and the presence of xenobiotics is noteworthy, as its activity can be a sensitive indicator of response to pollutants and contaminants, such as heavy metals or toxic chemicals [59]. It is generally observed that when aquatic organisms are exposed to pollutants, CAT activity often increases as an antioxidant defense strategy, aiming to neutralize the harmful effects of free radicals generated by oxidative stress induced by contaminants. This, in turn, contributes to assessing the health of aquatic ecosystems and water quality as a potential biomarker [60]. Moreover, oxidative enzymes such as superoxide dismutase, CAT, glutathione‐S‐transferase, lipid peroxidation, and GSH peroxidase are crucial biochemical markers for assessing metal toxicity in open water systems [61].

The evaluation of reduced GSH levels (Figure 5B) showed an increase in April and May, which contrasts with the behavior observed for CAT. High levels of CAT activity (Figure 5C) may be related to environmental stress and the biotransformation effort of pollutants, as observed in the species *Cottus gobio* [62], and also due to the influence of Cu concentration present in the water. Studies conducted with *Litopenaeus vannamei* have shown that the presence of this metal affects CAT activity [63]. The increase in Cu levels in April may be associated with a decrease in CAT enzyme activity during the same sampling period. This occurs because Cu has the ability to bind to this enzyme, altering its secondary structure and consequently causing a loss of activity [64]. The same effect was observed in studies with crustaceans of the *genus Aegla* [11]. It is important to emphasize that CAT is a peroxisomal enzyme capable of converting H_2_O_2_ into water and oxygen, and it is considered an early and reliable biomarker of contamination [56].

The increase in GSH activity may be related to a deficiency in CAT, which can be compensated by the activation of antioxidant defenses measured through GSH [11]. In studies conducted with *Callinectes amnicola*, it was found that GSH levels in ovarian tissues were higher, and during dry seasons, these levels were even greater [65]. CAT activity and TBARS levels were negatively correlated with Cu concentration in water in other studies [66], as well as in the species *Galleria mellonella*, where such exposure caused drastic effects, leading to delayed larval development and a shortened lifespan [67]. These seasonal variations highlight the dynamic interaction of antioxidant responses in aquatic organisms exposed to fluctuating environmental stressors [68], influencing reduced GSH levels and CAT activity, with GSH acting to protect against oxidative damage. Furthermore, studies with *Neomysis awatschensis* revealed that GSH activities increased significantly in adult mysids exposed to metals, including Cu [69], which can also be observed in our study. In contrast, CAT did not show a significant correlation with Cu throughout the sampling period, indicating that the enzymatic pathway associated with H_2_O_2_ degradation was not strongly activated under the observed conditions. However, a reduction in catalytic activity was noted, a pattern also observed in studies with *Moina macrocopa* [70].

The changes in GSH, CAT, Cu, and STD levels observed suggest that the crustacean *M. amazonicum* is a potential bioindicator and ecotoxicological biomonitor, which could be used to assess pollutants in aquatic areas. The positive correlations observed between Cu concentrations and GSH suggest a mechanistic relationship. Copper can participate in Fenton‐like reactions, increasing ROS generation, which stimulates the activation of antioxidant defenses such as GSH synthesis to mitigate oxidative damage [71]. In parallel, higher STD may enhance metal transport and bioavailability, potentially increasing Cu exposure in benthic organisms like *M. amazonicum* . Therefore, the concomitant patterns observed among these variables likely reflect biological responses to contaminant‐induced oxidative stress, rather than random associations. However, further studies are needed to better correlate the levels of transition metals with oxidative stress in the studied species and to confirm that the EPA is suffering oxidative damage from external pollutants.

This study has some limitations that should be acknowledged. First, metal concentrations were not measured directly in shrimp tissues, so inferences about bioaccumulation are based on water quality data and biomarker responses rather than direct tissue analysis. Future work should include tissue metal measurements to strengthen the mechanistic interpretation of biomarker responses. Second, sampling was restricted to the wet season. Seasonal variations, particularly during the dry season, can affect contaminant dynamics and oxidative stress patterns. Expanding sampling to include multiple seasons will provide a more comprehensive understanding of environmental stressors acting on *M. amazonicum* . Despite these limitations, the present study provides valuable baseline data and highlights the relevance of oxidative stress biomarkers for biomonitoring in Amazonian conservation areas.

## 4 Conclusions

The results indicate that the parameters of pH, temperature, Fe, and STD are within the standards established by CONAMA/BRAZIL 357/2005, while the Cu analyses are not. Furthermore, it is observed that high concentrations of this metal, as the methodology used demonstrates more favorable results for Cu analysis, can induce the species *M. amazonicum* to undergo alterations in oxidative stress biomarkers such as CAT and GSH. The biomarkers of CAT and TBARS are inversely related, as is the relationship between CAT and GSH. Thus, the inferred exposure to Cu, supported by water concentrations and antioxidant biomarker responses, explains the observed increases in GSH and CAT activity, indicating that the presence of this metal induces some level of oxidative stress in this species. These results highlight the importance of monitoring water quality in environmental conservation areas, especially concerning the presence of heavy metals such as Cu, which can adversely affect aquatic organisms. The use of oxidative stress biomarkers such as CAT and GSH has proven to be an effective tool for assessing the impacts of contamination on the health of aquatic ecosystems.

## Author Contributions

Conceptualization: G.A.‐S., N.E.S.; Methodology: A.J.S.D., L.V.S.S., N.M.A.; Data Collection: N.E.S.; Statistical Analysis: J.R.D.L.; Investigation: N.E.S., A.J.S.D.; Writing – Original Draft Preparation: N.E.S.; Writing – Review and Editing: J.R.D.L., G.A.‐S., D.C.S.; Supervision: G.A.‐S. All authors read and approved the final version of the manuscript.

## Consent

The authors have nothing to report.

## Conflicts of Interest

The authors declare no conflicts of interest.

## Data Availability

The data that support the findings of this study are available from the corresponding author upon reasonable request.
